# Development and characterization of a saturated transposon mutant library of *Salmonella enterica* serovar Enteritidis LA5

**DOI:** 10.1128/mra.01319-24

**Published:** 2025-05-27

**Authors:** Jérôme Trotereau, Anne-Sophie Huguet, Romain Jouan, Florent Kempf, Delphine Naquin, Catherine Schouler, Philippe Velge, Peter Mergaert, Isabelle Virlogeux-Payant

**Affiliations:** 1 INRAE, Université de Tours, ISP, Tours, Centre-Val de Loire, France; 2 Université Paris-Saclay, CEA, CNRS, Institute for Integrative Biology of the Cell, Gif-sur-Yvette, Île-de-France, France; University of Maryland School of Medicine, Baltimore, Maryland, USA

**Keywords:** *Salmonella *Enteritidis, Tn-seq, transposons, virulence plasmid, essential genes, TRANSIT, growth advantage, growth disadvantage

## Abstract

We created and characterized a saturated Tn-seq mutant library in *Salmonella enterica* subsp. *enterica* ser. Enteritidis strain LA5. The set of essential genes after culture in rich medium was determined. This functional genomics tool will aid in identifying the roles of chromosomal and plasmid virulence determinants.

## ANNOUNCEMENT

*Salmonella enterica* subsp. *enterica* ser. Enteritidis is one of the most commonly isolated *Salmonella* serovars in humans (1), causing diseases like gastroenteritis in humans, lethal systemic infections in mice, and remaining asymptomatic in poultry (2–4). One promising approach to studying pathogens is the use of transposon-saturated libraries coupled with high-throughput sequencing (Tn-seq). To implement this approach, we constructed a library using the *S*. Enteritidis LA5 strain, a phage type 4 wild-type field isolate from infected chickens widely characterized in various *in vitro* and *in vivo* models by the research community (5–9).

**Fig 1:**
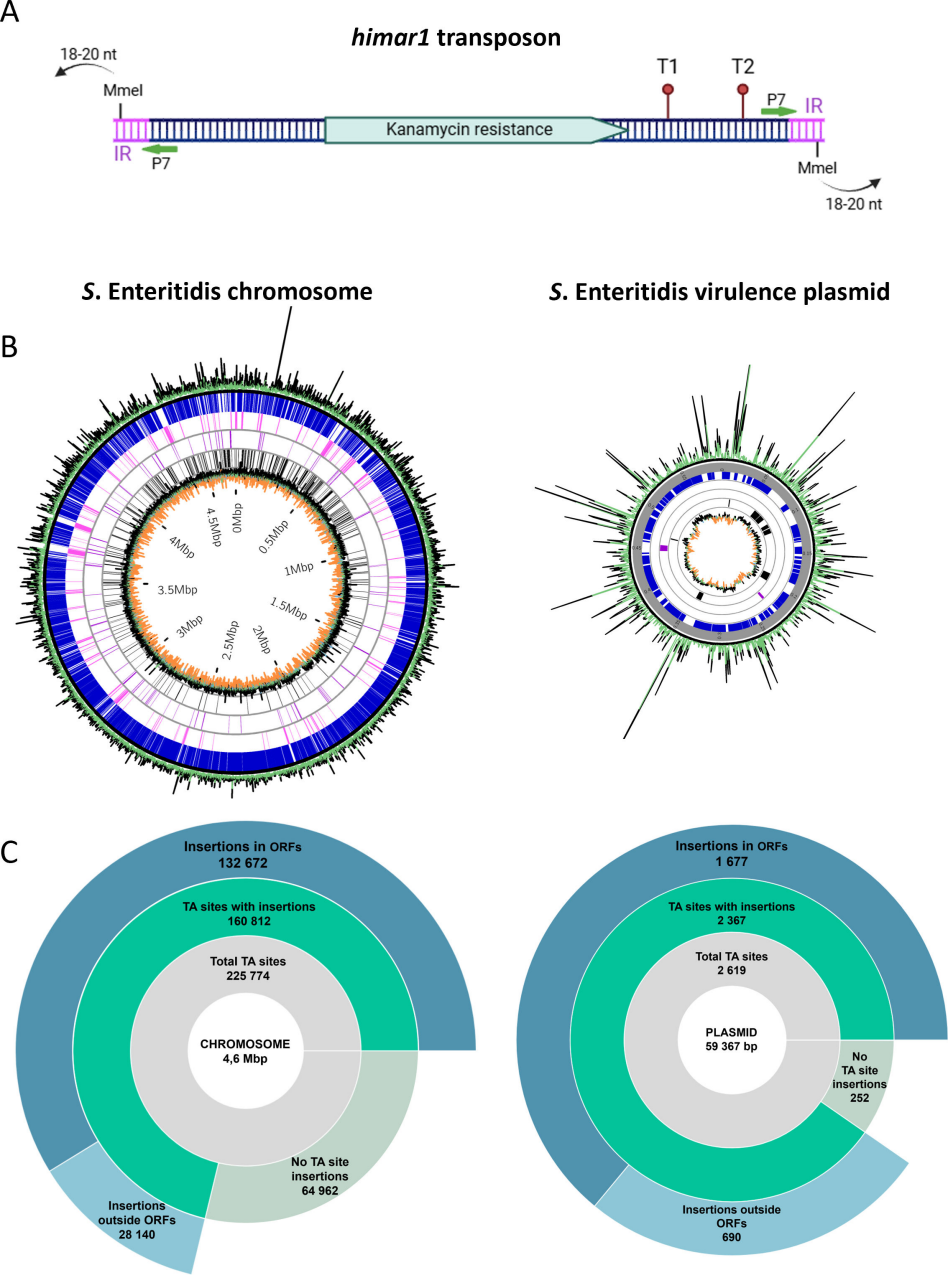
(**A**) Representative scheme of the *himar1* transposon. Inverted repeat (IR) sequences are shown in purple, with the MmeI recognition site located within them. Arrows indicate that MmeI cleavage sites are outside the transposon. The regions where primers P7 hybridize are marked with green arrows. Transcription terminators T1 and T2, corresponding to rrnB T1 and rrnB T2, are represented as red schematic symbols. (**B**) Circular representation of the Tn-seq library insertions in the *S*. Enteritidis genome, generated using the CIRCOS software (17). Both the chromosome and the virulence plasmid are saturated with insertions, depicted as green and black lines, respectively, with the latter being above the average. Through the use of TRANSIT software, we have identified various types of genes: non-essential genes (blue lines), essential genes (pink lines), genes with insertion resulting in a growth defect (purple lines), and/or a growth advantage (black lines). The GC percent of the genome is visualized by black, green, and orange colors on the innermost circle, representing below average, around average, and above average GC content, respectively. (**C**) The number of TA sites and the distribution of insertions, both within and outside the ORFs, are presented.

We used a *Himar1* mariner transposon (Fig. 1A) (10), which inserts into TA sequences. The LA5 genome contains 225,774 and 2,619 TA sites in its chromosome and plasmid, respectively, enabling the use of the Himar1 transposon to generate a saturated library. Library construction was performed as previously described (11, 12). Briefly, conjugation between *E. coli* MFDpir (pSAM_EC) and *S.* Enteritidis LA5 was performed at 37°C on tryptic soy agar (TSA) plates with 300 µg/mL DAP. After 12 hours, transconjugants were enumerated to assess library complexity, and the rest were plated on 400 TSA plates with 50 µg/mL kanamycin, without DAP, to expand the library for 18 hours at 37°C. The library yielded 2 × 10⁶ clones, which were pooled and stored at −80°C in 25% glycerol. Mutant DNA was extracted (Microbial DNA Mini Kit, Macherey-Nagel), processed (restriction, dephosphorylation, adaptor ligation, PCR), and sequenced (75 bp single-read, Illumina NextSeq 550, I2BC, CNRS). A total of 20,111,527 reads (110× coverage) were mapped to TA sites in the *S*. Enteritidis LA5 775 complete genome, which is a spontaneous streptomycin-resistant mutant of LA5 (K42T mutation in *rpsL*). Adaptor primers are listed in Table 1. The FastQC (FastQC v0.11.9, available online at: http://www.bioinformatics.babraham.ac.uk/projects/fastqc/) results are as expected, that is, with quality declining after 50 bases due to adapter sequencing. Barcodes were removed using Cutadapt 3.5 (13), transposons eliminated, and sequences filtered to 14–18 bases using SeqKit 2.0.0 (14). Sequences were reverse-complemented, and only those starting with “TA” were selected. The processed sequences were aligned to the LA5 775 complete genome using Bowtie 1.3.1 (15) with no mismatches allowed and only one alignment per read. Finally, a Wiggle file was generated. The cleaned and aligned data were analyzed using TRANSIT v3.3.8 (16), as described previously (11), to identify essential genes and those whose disruption confers a growth advantage or a disadvantage to *Salmonella* Enteritidis on TSA agar. Quantile normalization was applied to read counts to equalize the distribution of insertion reads across all sites, minimizing biases caused by technical variability or over-represented regions. A total of 160,812 TA sites with insertions were identified on the chromosome, and 2,367 on the plasmid in the *S*. Enteritidis LA5 Tn-seq library, with an average insertion every 30 base pairs (Fig. 1B). No mutants were detected in four chromosomal ORFs (RUI05_RS05600, RUI05_RS08405, RUI05_RS09135, and RUI05_RS20470) and one plasmid gene (RUI05_RS22785) due to the absence of TA sites and exclusion of the first and last 10% of each gene from TRANSIT analysis.

**TABLE 1:** Primers and adaptors used for library construction^*a*^

Name	Sequence from 5′ to 3′	Type	Target (or function)	Reference
Bu136	TTCCCTACACGACGCTCTTCCGATCTCAAGANN	Adaptor	Bu136 and Bu137 form an adaptor containing the barcode for sample multiplexing	(10)
Bu137	P-TCTTGAGATCGGAAGAGCGTCG TGTAGGGAAAGAGT-P	Adaptor		

^*a*^ P-, 5′ or 3′ phosphate; underlined sequence corresponds to the barcode.

On the chromosome, we identified 332 essential genes, as well as 223 and 292 genes whose disruption confers growth disadvantage or advantage on TSA plates, respectively. On the pSEV virulence plasmid, we found 2 and 10 genes with insertion-related growth disadvantage or growth advantage on TSA plates, respectively (Fig. 1C).

This Tn-seq mutant library in *Salmonella* Enteritidis LA5 will enable researchers to identify genetic determinants important in various conditions, thus allowing the study of pathogenicity in various hosts. The library is available upon request from the corresponding author.

## Data Availability

Raw sequencing data are accessible via the ENA website under the following accession number https://www.ebi.ac.uk/ena/browser/view/ERX10987239. Data on essential genes or genes whose disruption confers growth advantage or disadvantage to the S. Enteritidis LA5 strain are available at https://doi.org/10.57745/PIHAMK. The genome of the LA5 775 strain used is accessible under the accession number PRJNA1023056 via NCBI BioProject.

## References

[1] European Food Safety Authority and European Centre for Disease Prevention and Control. 2022. The european union one health 2021 zoonoses report. EFSA journal. doi:10.2903/j.efsa.2022.7666 PMC9745727 36524203

[2] Bäumler AJ, Tsolis RM, Ficht TA, Adams LG. 1998. Evolution of host adaptation in Salmonella enterica. Infect Immun 66:4579–4587. doi:10.1128/IAI.66.10.4579-4587.1998 9746553 PMC108564

[3] Mancin M, Barco L, Losasso C, Belluco S, Cibin V, Mazzucato M, Bilei S, Carullo MR, Decastelli L, Di Giannatale E, D’Incau M, Goffredo E, Lollai S, Piraino C, Scuota S, Staffolani M, Tagliabue S, Ricci A. 2018. Salmonella serovar distribution from non-human sources in Italy; results from the IT-Enter-Vet network. Vet Rec 183:69–69. doi:10.1136/vr.104907 29980593

[4] Uzzau S, Brown DJ, Wallis T, Rubino S, Leori G, Bernard S, Casadesús J, Platt DJ, Olsen JE. 2000. Host adapted serotypes of Salmonella enterica. Epidemiol Infect 125:229–255. doi:10.1017/s0950268899004379 11117946 PMC2869595

[5] Allen-Vercoe E, Dibb-Fuller M, Thorns CJ, Woodward MJ. 1997. SEF17 fimbriae are essential for the convoluted colonial morphology of Salmonella enteritidis. FEMS Microbiol Lett 153:33–42. doi:10.1111/j.1574-6968.1997.tb10460.x 9252570

[6] Cazals A, Rau A, Estellé J, Bruneau N, Coville J-L, Menanteau P, Rossignol M-N, Jardet D, Bevilacqua C, Bed’Hom B, Velge P, Calenge F. 2022. Comparative analysis of the caecal tonsil transcriptome in two chicken lines experimentally infected with Salmonella Enteritidis. PLoS One 17:e0270012. doi:10.1371/journal.pone.0270012 35976909 PMC9384989

[7] Kempf F, Menanteau P, Rychlik I, Kubasová T, Trotereau J, Virlogeux-Payant I, Schaeffer S, Schouler C, Drumo R, Guitton E, Velge P. 2020. Gut microbiota composition before infection determines the Salmonella super- and low-shedder phenotypes in chicken. Microb Biotechnol 13:1611–1630. doi:10.1111/1751-7915.13621 32639676 PMC7415355

[8] Naughton PJ, Grant G, Bardocz S, Allen-Vercoe E, Woodward MJ, Pusztai A. 2001. Expression of type 1 fimbriae (SEF 21) of Salmonella enterica serotype enteritidis in the early colonisation of the rat intestine. J Med Microbiol 50:191–197. doi:10.1099/0022-1317-50-2-191 11211228

[9] Ochoa-Repáraz J, Sebastià E, Sitjà M, Tamayo I, Irache JM, Gamazo C. 2021. Protection conferred by drinking water administration of a nanoparticle-based vaccine against Salmonella enteritidis in hens. Vaccines (Basel) 9:216. doi:10.3390/vaccines9030216 33802556 PMC8001700

[10] Lampe DJ, Akerley BJ, Rubin EJ, Mekalanos JJ, Robertson HM. 1999. Hyperactive transposase mutants of the Himar1 mariner transposon. Proc Natl Acad Sci USA 96:11428–11433. doi:10.1073/pnas.96.20.11428 10500193 PMC18050

[11] Trotereau J, Jouan R, Naquin D, Branger M, Schouler C, Velge P, Mergaert P, Virlogeux-Payant I. 2023. Construction and characterization of a saturated Tn-seq library of Salmonella Typhimurium ATCC 14028. Microbiol Resour Announc 12:e0036523. doi:10.1128/MRA.00365-23 37795997 PMC10653002

[12] Wallner A, Busset N, Lachat J, Guigard L, King E, Rimbault I, Mergaert P, Bena G, Moulin L. 2022. Differential genetic strategies of Burkholderia vietnamiensis and Paraburkholderia kururiensis for root colonization of Oryza sativa subsp. japonica and O. sativa subsp. indica, as Revealed by Transposon Mutagenesis Sequencing. Appl Environ Microbiol:e0064222. doi:10.1101/2022.04.14.488431 35862731 PMC9317867

[13] Martin M. 2011. Cutadapt removes adapter sequences from high-throughput sequencing reads. EMBnet j 17:10. doi:10.14806/ej.17.1.200

[14] Shen W, Le S, Li Y, Hu F. 2016. SeqKit: a cross-platform and ultrafast toolkit for FASTA/Q file manipulation. PLoS One 11:e0163962. doi:10.1371/journal.pone.0163962 27706213 PMC5051824

[15] Langmead B, Trapnell C, Pop M, Salzberg SL. 2009. Ultrafast and memory-efficient alignment of short DNA sequences to the human genome. Genome Biol 10:R25. doi:10.1186/gb-2009-10-3-r25 19261174 PMC2690996

[16] DeJesus MA, Ambadipudi C, Baker R, Sassetti C, Ioerger TR. 2015. TRANSIT--a software tool for himar1 TnSeq analysis. PLoS Comput Biol 11:e1004401. doi:10.1371/journal.pcbi.1004401 26447887 PMC4598096

[17] Krzywinski M, Schein J, Birol I, Connors J, Gascoyne R, Horsman D, Jones SJ, Marra MA. 2009. Circos: an information aesthetic for comparative genomics. Genome Res 19:1639–1645. doi:10.1101/gr.092759.109 19541911 PMC2752132

